# Enhancement of *Bacillus subtilis* Lipopeptide Biosurfactants Production through Optimization of Medium Composition and Adequate Control of Aeration

**DOI:** 10.4061/2011/653654

**Published:** 2011-09-27

**Authors:** Dhouha Ghribi, Semia Ellouze-Chaabouni

**Affiliations:** ^1^Départment de Biotechnolgie, Institut Supérieur de Biotechnologie de Sfax, B.P. 261, Sfax 3038, Tunisia; ^2^Unité Enzyme et Bioconversion, ENIS, B.P. W, Sfax 3038, Tunisia

## Abstract

Interest in biosurfactants has increased considerably in recent years, as they are potentially used in many commercial applications in petroleum, pharmaceuticals, biomedical, and food processing industries. Since improvement of their production was of great importance to reduce the final coast, cultural conditions were analyzed to optimize biosurfactants production from *Bacillus subtilis* SPB1 strain. A high yield of biosurfactants was obtained from a culture of *B. subtilis* using carbohydrate substrate as a carbon source; among carbohydrates, glucose enhanced the best surfactin production. The optimum glucose concentration was 40 g/L. Higher amount of biosurfactants was obtained using 5 g/L of urea as organic nitrogen source and applying C/N ratio of 7 with ammonium chloride as inorganic nitrogen source. The highest amount of biosurfactants was recorded with the addition of 2% kerosene. Moreover, it was shown, using an automated full-controlled 2.6 L fermenter, that aeration of the medium, which affected strongly the growth regulated biosurfactants synthesis by the producing cell. So that, low or high aerations lead to a decrease of biosurfactants synthesis yields. It was found that when using dissolved oxygen saturation of the medium at 30%, biosurfactants production reached 4.92 g/L.

## 1. Introduction

Biosurfactants are biologically surface active compounds produced by several microorganisms. In recent years, interest in biosurfactants has generated due to their possible applications in environmental protection, crude oil drilling, and in the pharmaceutical and food processing industries [1]. They acquired their importance from their multiples properties such as soaping, emulsifying, foaming, and dispersing [2].

Chemically synthesized surfactants have been used in the oil industry to aid clean-up of oil spills, as well as to enhance oil recovery from oil reservoirs. These compounds are not biodegradable and can be toxic to environment. Biosurfactant have special advantage over their commercially manufactured counterparts because of their lower toxicity, biodegradable nature, and effectiveness at extreme temperature, pH, salinity, and ease of synthesis [2, 3]. They were efficiently used in handling industrial emulsions, control of oil spills, biodegradation and detoxification of industrial effluents, and in bioremediation of contaminated soil [3, 4].

Several *Bacillus *species produce a lipopeptide biosurfactants; the most important one is surfactin which is produced from *Bacillus subtilis* [5, 6]. Surfactin is not ribosomally synthesized; it is synthesised by a multifunctional enzyme system as that involved in the synthesis of the peptide antibiotics released from bacilli bacteria [5]. Moreover, *B. licheniformis *has the ability to produce many surface active lipopeptides [7, 8].

In order to achieve high production of such biologically surface active compound from the newly isolated *Bacillus subtilis*, monitoring the key fermentation parameters (biomass, substrate, C/N ratio, aeration, etc.) is necessary. In this paper, we optimized the physicochemical parameters for surfactin high-level production. Large-scale production of surfactin was achieved in an automated, fully controlled fermenter.

## 2. Materials and Methods

### 2.1. Microorganisms

Biosurfactant producing bacterium was isolated in our laboratory from Tunisian soil according to the method described by Carrera et al. [9]. It was identified as *B. subtilis *SPB1 by morphological, biochemical, and 16S rDNA sequence analysis. The Gene-Bank accession number for the nucleotide sequence is HQ392822. This strain was used for biosurfactant production studies because of its large spectrum of bioactivity which have a great potential for biotechnological and biopharmaceutical applications. 

### 2.2. Inoculum and Culture Conditions

The inocula were prepared as following: one isolated colony was dispensed in 3 mL of LB medium and incubated overnight at 37°C. Aliquots (0.2 mL) were used to inoculate 250 mL Erlenmeyer flasks containing 50 mL LB medium [10] and incubated in a rotatory shaker at 200 rpm and 37°C (±0.5) until absorbance around 3, measured spectrophotometrically at 600 nm was reached. The culture broth was used to inoculate the studied media to start with an initial optical density of 0.1, corresponding to almost 8 × 10^7^ CFU/mL, except particular indications in Results and Discussion Sections.

Production medium was composed of basal salts containing (g/L) KH_2_PO_4_ (0.5), K_2_HPO_4 _(1), KCl (0.1), MgSO_4_ (0.5), FeSO_4_ (0.008), CaCl_2_ (0.05), Urea (6) with the addition of 1 ml/L trace elements solution (4.4 mg/L ZnSO4, 3.3 mg/L MnSO4, 0.1 mg/L CuSO4 and 1 mg/L NaBr) at pH 7, and supplemented with different carbon and nitrous sources at concentrations mentioned with results. 

 Samples were collected at time-defined intervals and submitted to analysis for determination of biomass production, glucose consumption, and changes in biosurfactants concentration. All experiments were performed in triplicate.

### 2.3. Biomass Determination

Sample was taken at regular interval and the number of cells was estimated by counting colony forming units (CFU). Appropriate dilutions of culture samples were plated on solid LB medium and incubated at 37°C overnight. The values presented are the average of the results of three determinations of two separate experiments for each cultural condition. 

### 2.4. Extraction of Crude Biosurfactant

The crude biosurfactant was isolated from the cell free broth of 48 h grown culture. The bacterial cells were removed from surfactant containing culture broth by centrifugation at 10.000 rpm at 4°C for 20 min. The supernatant was precipitated overnight at 4°C by adding concentrated HCl to achieve a final pH of 2.0, to precipitate lipids and proteins [11, 12]. Grey white pellets formed by precipitation were collected by centrifugation at 10.000 rpm at 4°C for 20 min. The crude surfactant was lyophilized and weighted for quantification. For the extraction of biosurfactant compounds, 50 mL of chloroform methanol (2 : 1v/v) was added to 500 mg of the dry product and incubated in a rotatory shaker at 250 rpm, 30°C (±0.5) for 15 minutes. The extract was evaporated to dryness and weighted for quantification. Assays were carried out in triplicates.

### 2.5. Determination of Emulsification Activity

Samples (0.5 mL) of cell-free supernatant were added to a screw-capped tube containing 7.5 mL of Tris-Mg (20 mM Tris HCl (pH 7.0) and 10 mM MgSO4) and 0.1 mL of kerosene. After a vigorous vortex, the tubes were allowed to sit for 1 hour. Absorbance was measured at 540 nm [2]. Emulsification activity (EA) was defined as the measured optical density. Assays were carried out in triplicates.

### 2.6. Biosurfactant Production into 2.6 L Fermenter

Production experiments were carried out at 37°C in 2.6 L labfors full controlled fermenter (Infors, Switzerland) containing 1.6 L of culture medium with continuous regulation of pH using 2 N HCl and 2 N NaOH. Dissolved oxygen level in the medium was automatically controlled by control of agitation and air flow as described in the Results Section. Dissolved oxygen was continuously monitored by an oxygen sensor (InPro 6000 Oxygen sensor, Mettler Toledo, Switzerland). Foaming was controlled by the use of an antifoam (Struktol SB2020, Schills seilacher, Hamburg, Germany), throughout the fermentation [13, 14].

### 2.7. Statistical Analysis of Results

All the results related to determination of emulsification activity, biosurfactants quantity and CFU counts were the average of three replicates of two separate experiments for each cultural condition. They were statistically analyzed by SPSS software (version 100) using the Duncan test performed after analysis of variance (ANOVA).

## 3. Results 

### 3.1. Effect of the Carbon Source on Biosurfactants Production

To select the suitable carbon nutrient supplements which would affect the composition of produced biosurfactants [15], substrates such as glucose, sucrose, starch, and glycerol were tested for the production of biosurfactants by *Bacillus subtilis* SPB1 strain. Indeed, the strain was able to use all these substrates; but the use of glucose as carbon source to produce biosurfactants seems to be more interesting. The bacterium produced 720 mg/L of biosurfactants at the end of the fermentation (data not shown). This result was expected since this carbon source is taken up more easily than compared to the others.

**Figure 1:**
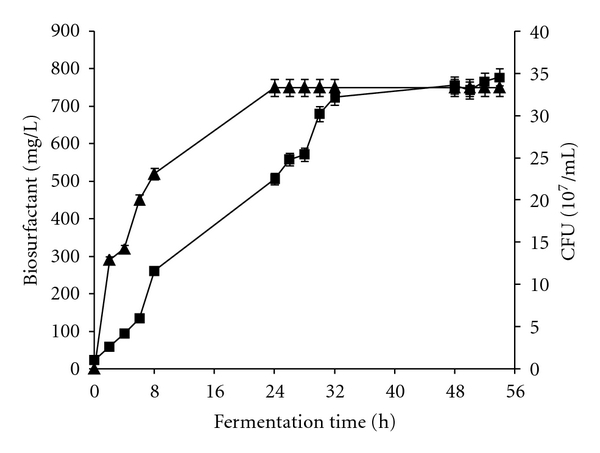
Kinetics of growth (■) and biosurfactants production (▲) by *Bacillus subtilis* SPB1 strain using the initial basal salt medium.

The microbial growth kinetics and biosurfactants production in the fermentation with 4% concentration of glucose are represented in Figure 1. The stationary phase was reached after 28 hours of fermentation, while optimal biosurfactants production was reached until 24 hours of growth. The specific growth rate was evaluated to 0.2772 h^−1^. 

Since the production medium was optimized with glucose as sole carbon source, different concentrations of glucose were examined for the best yield of biosurfactants from the studied strains. Glucose was added to the production medium in concentrations 15, 20, 25, 30, 35, 40, and 45 g/L, the results obtained (Table 1) showed a linear increase in, biosurfactants concentration with increasing the initial glucose concentration up to 40 g/L. Statistical analysis of the results based on Student and Duncan tests performed after ANOVA analysis showed that the highest biosurfactants production was obtained when using 40 g/L glucose. In such condition, biosurfactants production was evaluated to 720 mg/L corresponding to a specific yield (biosurfactant synthesis yield) of 25.71 mg/10^10^ CFU.

### 3.2. Effect of the Nitrogen Source on Biosurfactants Production

Since medium constituents other than carbon sources also affect the production of biosurfactants [16], *B. subtilis* SPB1 strain was cultivated, individually, with different organic nitrogen sources such as urea, pancreatic digest of casein, beef extract, yeast extract, or casein hydrolysate. The results obtained (Table 2) showed that the highest biosurfactants production determined in the culture broth (720 mg/L) was obtained when using urea as organic nitrogen source. It could be enhanced to 750 mg/L if the concentration of urea was only 5 g/L (data not shown).

**Figure 2:**
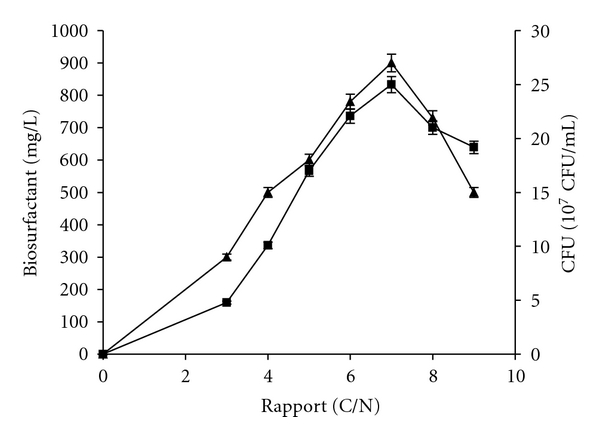
Effect of C/N ratio on biomass (■) and biosurfactants production (▲) by *Bacillus subtilis* SPB1 strain.

Inorganic nitrogen requirement of *B. subtilis SPB1* for biosurfactants synthesis was investigated through the variation of C/N ration which is strongly affected by the ammonium chloride (NH_4_Cl) concentration. Results of Figure 2 showed a significant increase of biosurfactants production up to C/N of 7 reaching 900 mg/L. Beyond such C/N ratio, a decrease of biosurfactants synthesis by produced cells was clear.

### 3.3. Effect of Hydrocarbons Addition on Biosurfactants Production

**Figure 3:**
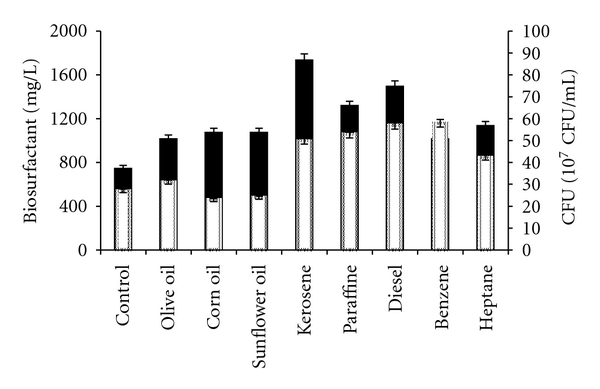
Effect the addition of oils or hydrocarbons on biomass (□) and biosurfactants production (■) by *Bacillus subtilis* SPB1 strain.

To improve biosurfactants production yield, different oils and hydrocarbons were added. Results of Figure 3 showed clearly that supplementing the optimized medium with 2% oils like olive oil, sunflower oil, corn oil, and paraffin or 2% hydrocarbons such as kerosene, diesel, benzene, and heptane could enhance biosurfactants production by SPB1 strain. The most important production was obtained when using the kerosene. It was evaluated to 1740 mg/L. Indeed, the assimilation of such hydrocarbon clearly improved biosurfactants synthesis yield by SPB1 cells reaching 34.80 mg/10^10^ CFU corresponding to an improvement factor of 30%.

### 3.4. Effect of Inoculum Size on Biosurfactants Production

**Figure 4:**
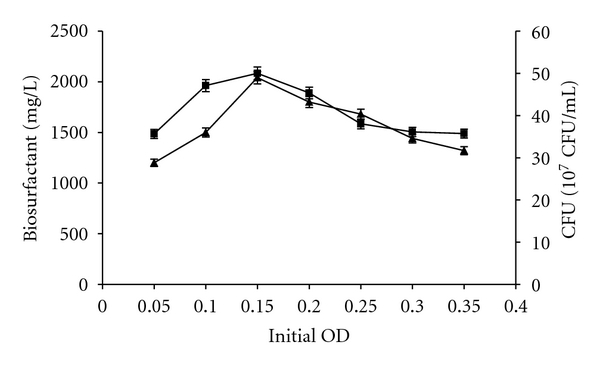
Effect of the inoculum size estimated by the initial OD_600_ of the culture on biomass (■) and biosurfactants production (▲) by *Bacillus subtilis* SPB1 strain.

When the strain was inoculated into the production medium, the inoculum size was adjusted by adjusting initial OD_600_ from of 0.05 to 0.35. The data provided in Figure 4 showed that the initial OD_600_ less or more than 0.15 resulted in a low level of biosurfactants yield, indicating that the optimum of biosurfactants production would be obtained by starting the culture of *B. subtilis* SPB1 strain with an initial OD_600_ of 0.15. In such condition, biosurfactants production reached 2040 mg/L.

### 3.5. Effect of the New Optimized Medium on Growth and Biosurfactants Production by SPB1 Strain

**Figure 5:**
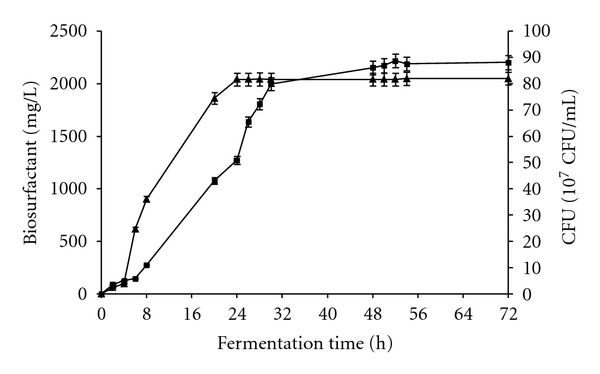
Kinetics of growth (■) and biosurfactants production (▲) by *Bacillus subtilis* SPB1 strain using the new formulated medium.

Kinetics of growth and production of biosurfactants by *Bacillus subtilis *SPB1strain were studied using the new optimized parameters (Figure 5). It was clear that, compared to results obtained using the initial medium, productivity and biosurfactants synthesis yield were increased under these new conditions reaching, respectively, 85 mg/L/h and 25.37 mg/10^10^ CFU, while the growth rate fall down to 0.173 h^−1^. These results showed that the new optimized conditions are in favour of the increase of the generation time of SPB1 strain allowing more time to produce biosurfactants which was shown to be a growth-associated metabolite.

### 3.6. Investigation of the Dissolved Oxygen Requirement for Biosurfactants Production by Bacillus subtilis SPB1 Strain at 2.6 L Fermenter Scale 

Since control of dissolved oxygen was necessary for regulating the carbon source assimilation rate and consequently the metabolites synthesis [13, 14], the dissolved oxygen requirement for biosurfactants production by *Bacillus subtilis* SPB1 strain, in the optimized medium was evaluated in an automated full-controlled 2.6 L fermenter. To elucidate the implication of oxidative metabolism in biosurfactants production, different aeration profiles were used throughout the fermentation. The obtained results (Table 3) showed that there was an increase in biosurfactants production with increasing the percentage of dissolved oxygen saturations up to 30%. In the later condition, both growth and biosurfactants production were improved. In such condition, biosurfactants production reached 4922.540 mg/L corresponding to a production yield of about 24.61 mg/10^10^ CFU. Meanwhile, biosurfactants production decreased with higher aerations. It was evaluated to 4230.423 mg/L with 40% of dissolved oxygen saturation and it was dramatically reduced with 60% of dissolved oxygen saturation (2609.140 mg/L).

## 4. Discussion

In order to reach overproduction of biosurfactants, nutritional requirements of *Bacillus subtilis *biosurfactant-producing strain and growth parameters were studied. As previously demonstrated by [15], different carbon sources could be used in the medium for biosurfactants production. *Bacillus subtilis* SPB1 strain was able to use substrates such as glucose, sucrose, starch, and glycerol to produce biosurfactants but the use of glucose as carbon source seems to be more interesting. The bacterium produced 720 mg/L of biosurfactants at the end of the fermentation. This result was expected since this carbon source is taken up more easily than compared to the others [17, 18]. Thus, different concentrations of glucose were examined for the best yield of biosurfactants production confirming that 40 g/L glucose was the optimal concentration for biosurfactants production. With higher glucose concentrations, biosurfactants production in the media was significantly decreased, but biomass continued to increase. This could be explained by the fact that the produced cells at high glucose concentrations are not physiologically adequate to synthesize biosurfactants [18]. On the other hand, it was clear that a balance between organic and inorganic nitrogen sources should be taken into account for biosurfactants synthesis by the produced cell biomass. Consequently, using adequate concentrations of urea as organic nitrogen source and applying adequate C/N ratio with ammonium chlorideas inorganic nitrogen source efficiently oriented the cell metabolism in favour of biosurfactants synthesis [19]. Moreover, due to the ability of biosurfactants to degrade aromatic compounds, addition of hydrocarbons into the culture medium-enhanced biosurfactants production [20].

It was also shown that adequate density of the inoculum or seed culture was determinant for high biosurfactants production [21]. It was well known that it could influence the duration of lag phase, specific growth rate, biomass yield, and quantity of the final product [14].

 Environmental factors and growth conditions such as agitation, and oxygen availability also affect biosurfactant production through their effect on cellular growth or activity [22]. This was confirmed when varying the percentage of dissolved oxygen into the media throughout the fermentation for biosurfactants production by *Bacillus subtilis* SPB1 at 2.6 L fermenter scale. Particularly, it was shown that aeration of the medium, which affected strongly the growth, regulated biosurfactants synthesis by the producing cell. Such results were also observed by Clarke et al. [23]. Low or high aerations lead to a decrease of biosurfactants synthesis yields confirming results obtained with Shepperd and Cooper [24] showing that oxygen transfer is one of the key parameters for the process, optimization, and scale up of biosurfactants production by *B. subtilis.*

## Figures and Tables

**Table 1:** Effect of the variation of glucose concentrations on biomass and biosurfactant production by *Bacillus subtilis* SPB1 strain.

Glucose (g/L)	Biosurfactants (mg/L)	CFU (10^7^ CFU/mL)	Sugar utilisation (%)	*Relative biosurfactants yield (mg/10^10^ CFU)
15	420 ± 5	18 ± 1	100	23.33
20	480 ± 8	20 ± 2	100	24.00
25	540 ± 10	22 ± 1	100	24.54
30	600 ± 15	24 ± 2	100	25.00
35	660 ± 5	27 ± 2	100	24.44
40	720 ± 7	28 ± 1	99	25.71
45	690 ± 6	30 ± 3	97	23.00

*Relative biosurfactant yield was calculated as the ratio of biosurfactant (mg/L) over CFU (10^10^ CFU/L), both of them were determined at the end of fermentation.

**Table 2:** Effect of the variation of organic nitrogen source on biomass and biosurfactant production by *Bacillus subtilis* SPB1 strain.

Organic nitrogen source	Biosurfactants (mg/L)	Biomass (10^7^ CFU/mL)	*Relative biosurfactants yield (mg/10^10^ CFU)
Casein hydrolysate	630 ± 5	25 ± 1	25.20
Beef extract	600 ± 11	24 ± 3	25.00
Urea	720 ± 5	27 ± 2	26.66
Pancreatic digest of casein	660 ± 9	28 ± 3	23.57
Yeast extract	660 ± 10	27 ± 1	24.44

*Relative biosurfactant yield was calculated as the ratio of biosurfactant (mg/L) over CFU (10^10^ CFU/L), both of them were determined at the end of fermentation.

**Table 3:** Aeration effect on SPB1 biosurfactants production into 2,6 L fermentor using the formulated medium.

Aeration profile (%)	Biosurfactants (mg/L)	CFU (10^8^ cells/mL)	*Relative biosurfactants yield (mg/10^10^cells)
10	2410.160 ± 23	15.30 ± 2	15.75
20	3801.120 ± 34	16.60 ± 3	22.89
30	4922.540 ± 25	20.00 ± 1	24.61
40	4230.423 ± 13	27.50 ± 3	15.38
60	2609.140 ± 11	36.00 ± 1	7.24

*Relative biosurfactant yield was calculated as the ratio of biosurfactant (mg/L) over CFU (10^10^ CFU/L), both of them were determined at the end of fermentation.
